# Influence of physician networks on prescribing a new ingredient combination in heart failure: a longitudinal claim data-based study

**DOI:** 10.1186/s13012-021-01150-y

**Published:** 2021-08-28

**Authors:** Christine Arnold, Jan Koetsenruijter, Johanna Forstner, Frank Peters-Klimm, Michel Wensing

**Affiliations:** Department of General Practice and Health Services Research, Heidelberg University Hospital, Im Neuenheimer Feld 130.3, 69120 Heidelberg, Germany

**Keywords:** Medication prescribing, Chronic heart failure, Ambulatory care, Social network analysis, Implementation science

## Abstract

**Background:**

Since 2016, the combination of sacubitril/valsartan, which combines an angiotensin receptor and neprilysin inhibitor (ARNI), has been recommended in the guidelines for the treatment of heart failure. The adoption of new drugs may be influenced by collaboration and exchange between physicians. We aimed to determine whether characteristics of the professional networks of prescribing physicians were associated with the prescribing of ARNI in Germany.

**Methods:**

We conducted a longitudinal analysis based on claims data in 2016–2018 in Germany. The characteristics of ambulatory care physicians’ networks were determined in the analysis of the patient-sharing networks of physicians in 2017. Binary logistic regression analysis with the outcome ‘prescribes ARNI in 2018’ (present or absent) was carried out, using network characteristics as predictors, adjusted for specialty and sociodemographic characteristics of physicians.

**Results:**

The network analysis included 8370 physicians, who had 144,636 connections. Prescribers had more connections to other physicians compared to non-prescribers (median 31 vs. 23). Regression analysis showed that the numbers of linkages to prescribers of ARNI were positively associated with prescribing ARNI. For 6–10 connections, the average marginal effect (AME) was 0.04 (confidence interval [CI] 95% 0.01–0.06) and for > 10 links the AME 0.07 (CI 95% 0.05–0.10) compared to 0–5 connections to prescriber.

**Conclusion:**

Physicians who shared patients with many other physicians were more likely to prescribe ARNI, independent of physicians’ specialty. This suggested that collaboration and exchange on the basis of patient-sharing with other physicians influenced their medication prescribing decisions.

**Supplementary Information:**

The online version contains supplementary material available at 10.1186/s13012-021-01150-y.

Contributions to the literature

- This study provides quantitative estimations of the effects of patient-sharing networks on physicians’ uptake of a new drug, using large-scale claims data on real-world ambulatory care.
- The study showed that a higher number of connections with other prescribers of the new drug was associated with a higher probability of prescribing the drug.
- The network effects are small, but they exist at a large scale and are probably sustained over time.

## Background

In 2017, the prevalence of heart failure (HF) in Germany was 3.4% [1]. Due to increases in life expectancy and improved treatment, the prevalence of HF is expected to rise. Cardiovascular diseases, including HF, are among the most frequent causes of death worldwide [2]. Each year in Europe, cardiovascular diseases cause more than 4 million deaths—45% of all deaths [3]. Drug therapy is the most critical pillar in the treatment of HF. Since the treatment guidelines were updated in 2016, the European Society of Cardiology and the national guidelines in Germany have recommended the prescription of a combination of sacubitril and valsartan, if symptoms persist despite administration of basic therapy [4, 5]. Sacubitril/valsartan, which combines an angiotensin receptor and neprilysin inhibitor (ARNI), has been available in Germany since 2016 [6]. According to the data from statutory health insurance in Germany, 3.7 million defined daily dose (DDD) of ARNI were prescribed in the first year of approval, while in 2019, 34.1 million DDD were already documented [7, 8]. The PARADIGM-HF study demonstrated that ARNI reduces mortality due to HF more than the angiotensin-converting enzyme inhibitors enalapril [9]. Adoption of new medications, including ARNI, is often slow [10–12]. Wachter et al. [13] showed in their longitudinal cohort study that most patients did not receive the recommended dose for ARNI. To achieve the best possible treatment for patients, it is important to optimize the adoption of effective medicines.

Many factors influence the uptake of clinical practices among physicians, such as the prescribing of medication. In addition to patient factors and the benefit–harm profile of practices, individual physicians’ beliefs and routines play a role [14]. Their beliefs and routines may be particularly relevant if the practice is new, controversial (e.g. due to high cost), or associated with high uncertainty regarding clinical effects. Individual characteristics of physicians, such as sex, professional experience, and medical specialty designation, may influence the adoption of a new drug. For instance, cardiologists respectively specialists tend to adopt new drugs earlier than primary care physicians [14, 15]. Physicians’ beliefs and routines are also shaped by contextual influences, such as collaboration and exchange with peers [14, 15]. A systematic review by Lublóy [14] summarizes how social interactions among physicians were associated with the adoption of new drugs. The studies included in Lublóy’s review were predominantly from the USA and did not consider drugs used in cardiology. Donohue et al. [16] describes the influence of a peer network on the adoption of new drugs: a 10% increase in peer adoption in the patient-sharing network led to a 5.9% (standard error [SE] = 1.5%, *p* < 0.001) increase in physician adoption of dabigatran, a 8.3% (SE = 1.51%, *p* < 0.001) increase in adoption of sitagliptin, and a 7.84% (SE = 2.93%, *p* < 0.001) increase in adoption of aliskiren.

By treating common patients, it is likely that new recommendations will be adopted by physicians in line with social contagion. Social contagion is understood as the process in which one person adopts a new attitude or behaviour from another person either because the actor is directly influenced by them in the context of an interaction relationship (cohesion) or because the actor is in a structurally similar position within social networks (structural equivalence) [17].

In this study, we explored the role of collaboration and exchange between ambulatory care physicians in their prescription of sacubitril/valsartan, a new ingredient combination in Germany. In particular, we aimed to provide a quantitative estimate of the impact of physicians’ patient-sharing networks on the prescription of medication.

## Methods

### Study design

We conducted a longitudinal observational analysis based on health insurance claims data to test the influence of having professional relationships with physicians on prescriptions of ARNI in 2017. We used claims data from the health insurance company AOK (German: *Allgemeine Ortskrankenkasse*) Baden-Wurttemberg. This insurer covers approximately four million individuals, which is about 45% of the people living in the state Baden-Wurttemberg [18]. The pseudonymized data storage and extraction were performed with the software dbForge Studio for MySQL. Data analysis was conducted with the statistics software R version 3.6.1 using RStudio. The study received a positive ethics vote from the Ethics Committee of the Medical Faculty of Heidelberg (ID: S-726/2018). This study conforms to the standards for reporting observational studies (see Additional file 1).

### Study samples

The study population included those insured by the AOK and the principal physicians involved in their treatment who practise in Baden-Wurttemberg in the years 2017 and 2018. The physician population included the specialist groups that are primarily involved in the treatment of patients with HF (general practitioners, family doctors, internal medicine practitioners, cardiologists, pneumologists, angiologists, and nephrologists). In Germany, ambulatory specialist care is largely provided in office-based ambulatory practices. As the AOK covers the most people in Baden-Wurttemberg nearly all physicians in the region are included in this study.

### Network construction

Linkages between physicians were determined on the basis of shared patients within 12 months in 2017. From health insurance claims data, we extracted all physicians who worked in Baden-Wurttemberg and, based on the outpatient accounting number (German: *Einheitlicher Bewertungsmaßstab*), determined whether the same patient was treated by two physicians. This link is also called shared patient [19, 20]. The data was imported from MySQL into R and an overall undirected (i.e. links are by definition mutual) whole (all actors in a region are included) network was created using the package ‘igraph’. The nodes represent the physicians, who are involved in the treatment of HF, and the edges were linkages between the physicians based on at least five shared patients.

### Measures

#### Outcome

Prescription of ARNI in 2018 no (0) or yes (1) was extracted from health insurance claims data. A value of 1 was assigned if the physician prescribed ARNI for at least one of his/her patients

#### Predictor

Being linked to a prescriber of ARNI in 2017. This was extracted from the social network. Three categories were due to the distribution and the median created: few (0–5 links), medium (6–10 links), and many (> 10 links) connections to a prescriber.

*Covariates* were derived from the health insurance data or created through social network analysis. From the health insurance data, we obtained the *age* of the physicians in years, the sex (female and male), the *location* of the physician (rural ≤ 20,000 inhabitants and urban > 20,000 inhabitants), the *specialty* (family doctors, general practitioners, internal medicine, cardiologists, pneumologists, angiologists and nephrologists), and the participation in family doctor-centred care (German: *Hausarztzentrierte Versorgung*) as well as participation in provision of the disease management programme of chronic coronary heart disease. For adjustment in the regression analysis, we used the variable *prescription of ARNI 2017* dichotomously (0 = no and 1 = yes). We aggregated the number of treated heart failures (NYHA II–IV) patients per physician and summarized them into the metric variable ‘number of patients HF’.

The following variables were created through social network analysis.

#### Betweenness centrality

Betweenness centrality measures the proportion of pathways in the whole network for which the physician of interest is on the shortest path. There is a value range from 0 to 1. One means an actor is in all shortest paths of the network. The actor gets information faster compared to actors who are less often in the shortest path. Since the total network is very large and the variable therefore has very small values, we have multiplied the value by 1000.

#### Degree centrality

Degree measures the number of connections an actor has in the network. A high number of connections means a high degree centrality, which indicates that the physician has a central position in the network of shared patients.

#### Constraint

Constraint measures the extent to which time and energy is concentrated within a single cluster of connections [21]. A physician with low constraint is involved in many incomplete triads, so there are (many) structural holes in the network offering the physician options for brokering. A low constraint means that a physician can freely choose within the network where to get information and who to cooperate with. Values from 0 to 1 can be assumed. At 0, there is no limitation while at 1 full inclusion in the network is achieved by the maximum number of connections. For a better interpretation in the regression analysis, we multiplied the value by 10.

### Statistical analysis

First, we conducted descriptive analyses. This included frequencies and percentages of prescribing the new drug in the years 2016–2018. Depending on the scale level and distribution of the variables, frequencies in percentage, mean with SDs, and median with interquartile ranges were extracted. Chi-squared test or Wilcoxon’s rank-sum test was performed to detect differences in the groups of prescribers and non-prescribers in 2018.

Second, we carried out a social network analysis to identify social structures and characteristics that might explain the behaviour of doctors when prescribing a new drug. After the formation of the whole network with the statistic package ‘igraph’, the individual network properties of the physicians in the network were calculated. This included degree measures, which count the links of all physicians, betweenness centrality, and constraint. We have checked if there is a network constraint of 1 and at the same time only one connection to another physician. We assumed that these physicians were located on the outskirts of Baden-Wurttemberg and therefore a full network cannot be considered here and therefore their network properties cannot be measured reliably. These physicians (*n =* 96) were excluded from the further analysis.

Third, we carried out a binary multivariable logistic regression analysis with the dependent variable the prescription of ARNI in 2018. The main predictors were the categorial variable ‘links to prescriber of ARNI in 2017’ and the other network characteristics. The multivariable regression model was adjusted for other variables, such as sociodemographic data, specialist designation, or enrolment in a disease management programme for chronic coronary heart disease or family doctor-centred care centre, number of HF patients, and prescribing of ARNI in 2017. For easier interpretation, we present the results of logistic regression as average marginal effects. Due to the small number of missing values, we did not perform imputation. The independent variables were tested for multicollinearity and excluded if the correlation coefficient was greater than 0.6. We did not adjust the models for patient characteristics because we defined only the single prescription of ARNI as an outcome at the physician level and assumed that physicians have the option to prescribe the drug to at least one patient, regardless of patient characteristics. As a sensitivity analysis, we calculated a zero-inflated model with negative binomial distribution so that we could eliminate excess zeros due to a physician’s inability to prescribe the drug in the absence of heart failure patients. We also performed logistic regression with the subgroup of physicians who did not prescribe ARNI in 2017.

In this explorative study, we set a significance level of *α* = 0.05. We examined the impact of 10 predictors on the outcome.

## Results

The prescription of ARNI has continuously increased since its approval of access to the market in 2016. In the first year of approval (2016), 1127 (13.5%) physicians prescribed ARNI. Prescription of the new drug increased in the following years: in 2017, it was prescribed by 2110 (25.2%) physicians, and in 2018, it was prescribed by 3075 (36.5%) (see Table 1).

**Table 1:** Frequencies of insured users, heart failure diagnoses, and physicians

	2016	2017	2018
	*n* (%)	*n* (%)	*n* (%)
**Insured users of health services**	3,071,608	3,123,514	3,182,490
**Heart failure diagnosis (NYHA II–IV)**			
ICD-10 I50.12	50,257	55,637	59,178
ICD-10 I50.13	29,468	30,841	33,049
ICD-10 I50.14	5558	5888	6633
**Number of physicians**	8371	8370	8430
**Physicians prescribing ARNI**			
Yes	1127 (13.5)	2110 (25.2)	3075 (36.5)
No	7244 (86.5)	6230 (74.8)	5355 (63.5)

*NYHA* New York Heart Association, *ICD-10* 10th revision of the International Statistical Classification of Diseases and Related Health Problems, *ARNI* angiotensin receptor and neprilysin inhibitor

For the shared patients’ network in 2017, we initially identified 3,123,514 insured users of health services and 8370 ambulatory care physicians. These physicians had 144,636 connections between one another (that is, shared patients), 443 of whom were not active in Baden-Wurttemberg in 2018, leaving 7927 physicians with the main outcome prescription ARNI in 2018 and who shared at least five patients with other physicians. The largest group of physicians were family physicians with general practitioners, which together were 7071 (89.2%) physicians. Table 2 shows comparisons between prescribers and non-prescribers. While only 4.5% of cardiologists in 2017 were represented in the overall group of physicians, the number rose to 7.1% within the prescriber group.

**Table 2:** Characteristic in 2017 according to prescriber versus non-prescriber in 2018

	Total	Non-prescriber	Prescriber	***p***-value
	*N* = 7927	*n* = 4965 (62.6)	*n* = 2962 (37.4)	
	*n* (%)	*n* (%)	*n* (%)	
**Specialists**				< 0.001^a^
Family doctors	2266 (28.6)	1395 (28.1)	871 (29.4)	
General practitioners	4802 (60.6)	2970 (59.8)	1832 (61.9)	
Internal medicine	144 (1.8)	118 (2.4)	26 (0.9)	
Cardiologists	357 (4.5)	147 (3.0)	210 (7.1)	
Pneumologists	144 (1.8)	142 (2.9)	2 (0.1)	
Angiologists	34 (0.4)	31 (0.6)	3 (0.1)	
Nephrologists	180 (2.3)	162 (3.3)	18 (0.6)	
**Sex**				< 0.001^a^
Male	4884 (61.6)	2856 (57.5)	2028 (68.5)	
Female	3043 (38.4)	2109 (42.5)	934 (31.5)	
**Age**, years (mean, SD)	55.41 (9.0)	55.55 (8.9)	55.39 (9.1)	0.34^b^
**Location** (*n* = 7917)				< 0.001^a^
Rural	3501 (44.2)	2052 (41.4)	1449 (48.9)	
Urban	4416 (55.8)	2904 (58.6)	11512 (51.1)	
**Family doctor-centred care**				< 0.001^a^
No	4338 (54.7)	3057 (61.6)	1281 (43.2)	
Yes	3589 (45.3)	1908 (38.4)	1681 (56.8)	
**Disease management programme**				< 0.001^a^
No	1675 (21.1)	1393 (28.1)	282 (9.5)	
Yes	6252 (78.9)	3572 (71.9)	2680 (90.5)	

^a^Chi-squared test; ^b^Wilcoxon’s rank-sum test

After the exclusion of physicians with a constraint of 1, the network properties for 7831 physicians were calculated and included in the subsequent regression analysis. As previously described, we suspect that these physicians are located on the outskirts of Baden-Wurttemberg and no reliable network can be created, as no data outside of Baden-Wurttemberg was available. Table 3 shows the network characteristics subdivided according to prescription of ARNI in 2018. Prescribers in 2018 had more contacts with physicians who had already prescribed the new drug in 2017 as well as non-prescriber in the network. Non-prescribers in 2018 had a higher network constraint: median 0.08 (IQR 0.06) compared to prescriber median 0.06 (IQR 0.03). The prescribers exhibited a higher betweenness centrality median 0.03 (IQR 0.14) compared to the non-prescriber median 0.01 (IQR 0.08) in the network.

**Table 3:** Network characteristics in 2017 from prescriber versus non-prescriber in 2018

	Total	Non-prescriber	Prescriber	***p***-value
	*N* = 7831 (100%) (median, IQR)	*n* = 4873 (62.2%) (median, IQR)	*n* = 2958 (37.8%) (median, IQR)	
**Link to ARNI,***n* (%)				< 0.001^a^
Few (0–5 links)	3212 (41.0)	2342 (48.1)	870 (29.4)	
Medium (6–10 links)	2521 (32.2)	1431 (29.4)	1090 (36.9)	
Many (> 10 links)	2098 (26.8)	1100 (22.6)	998 (33.7)	
**Degree centrality**	27 (25)	23 (24)	31 (25)	< 0.001^b^
**Constraint**	0.07 (0.05)	0.08 (0.06)	0.06 (0.03)	< 0.001^b^
**Betweenness centrality**	0.02 (0.10)	0.01 (0.08)	0.03 (0.14)	< 0.001^b^

^a^Chi-squared test,^b^Wilcoxon’s test

*ARNI* angiotensin receptor and neprilysin inhibitor

*Degree* measures the number of connections an actor has in the network (values 0 to 7.830)

*Constraint* measures the extent to which time and energy is concentrated within a single cluster of connections (values 0 to 1)

*Betweenness centrality* measures the proportion of pathways in the whole network for which the physician of interest is on the shortest path (values 0 to 1)

Univariable and multivariable regression analyses showed no significant associations between prescribing and physician age. For this reason, we excluded age from the final model because it acted only as a covariate and no change in the explained variation was registered. The test for multicollinearity showed a very high correlation between the variables ‘link to ARNI prescriber’ and ‘degree centrality’ eta^2^ = 0.69. For this reason, we excluded degree centrality from the full model. Furthermore, constraint correlates negatively (eta^2^ = 0.56) with ‘link to ARNI prescriber’. The full final model shows an average marginal effect [AME] of 0.04 (confidence interval [CI] 95% 0.01–0.06), *p* < 0.01 for a medium physician’s connection and an AME of 0.07 (CI 95% 0.05–0.10), *p* < 0.001 for many links to an ARNI prescriber in comparison to few connections. In addition, constraint had an AME of − 0.05 (CI 95% − 0.07 to − 0.03), *p* < 0.001, which affects ARNI prescribing in 2018. The effects of betweenness centrality were not significant in the final model (see Table 4).

**Table 4:** Multivariable logistic regression dependent variable: ARNI prescription in 2018 (*N* = 7821)

Variable	AME	95% CI	***p***-value
Link to ARNI prescriber (ref. few 0–5 links)			
Medium (6–10 links)	0.04	0.01–0.06	< 0.01
Many (> 10 links)	0.07	0.05–0.10	< 0.001
Prescription ARNI 2017 (ref. no)	0.39	0.37–0.40	< 0.001
Constraint (multiplied by 10)	− 0.05	− 0.07 to − 0.03	< 0.001
Betweenness centrality (multiplied by 1000)	− 0.001	− 0.004 to 0.002	0.46
Specialists (ref. family doctors)			
General practitioners	− 0.005	− 0.02 to 0.02	0.63
Internal medicine	− 0.14	− 0.23 to − 0.05	0.001
Cardiologists	0.04	− 0.007 to 0.09	0.09
Pneumologists	− 0.46	− 0.68 to − 0.23	< 0.001
Angiologists	− 0.33	− 0.55 to − 0.10	< 0.01
Nephrologists	− 0.17	− 0.26 to − 0.08	< 0.001
Sex (ref. female)	0.03	0.01–0.05	< 0.01
Urban-rural (ref. rural)	− 0.04	− 0.06 to − 0.02	< 0.001
Family-doctors centred care (ref. no)	0.04	0.02–0.06	< 0.001
Disease management programme (ref. no)	0.08	0.05–0.11	< 0.001
Number of patients with heart failure	0.001	0.00–0.001	< 0.001
Total variance explained by the model (*R*^2^)	0.41	Cohen’s effect	0.84

*AME* average marginal effect, *CI* confidence interval

As a sensitivity analysis, we calculated a model without the network property constraint, since a correlation of eta^2^ = 0.56 of the variable’s constraint and ‘link to ARNI prescriber’ was shown. In this model, similar to the previously described model, medium links to ARNI prescriber show an AME 0.07 (CI 95% 0.05–0.09), *p* < 0.001, and many links to ARNI prescriber an AME 0.12 (CI 95% 0.09–0.14), *p* < 0.001, of being adjusted for the variables already described. In addition, we performed a subgroup analysis with only the 2017 non-prescribers, which confirmed our previous regression model results (see Additional file 2). A further sensitivity analysis with a zero-inflated model shows that the number of heart failure patients had a greater impact on the presence of excessive zeros than on prescribing behaviour.

## Discussion

Controlled for other factors, such as physicians’ specialty, this study found associations between professional networks of physicians and the adoption of a new drug. Physicians who have many connections to other physicians who already prescribed ARNI, who are well integrated into the patient-sharing network (high-degree centrality), and who have many connections to others who are not connected with each other (low constraint) were more likely to prescribe ARNI. Overall, the study suggests that exchange on the basis of shared patients influences physicians’ beliefs and routines regarding the prescription of medication.

Our study showed that prescriptions of the new drug combination (ARNI) are gradually increasing. This increase was also found in other studies [10–12, 22]. Previous studies [14] showed that specialists adopted new practices earlier than general practitioners. This is confirmed by our results for the new drug ARNI. In their qualitative study of prescribing behaviour after myocardial infarction, Freier et al. [23] describe how general practitioners feel uncertain about prescribing medications due to side effects, intolerances, comorbidities, and the number of guideline recommendations. This study also reported that general practitioners often ask cardiologists for advice and adopt the prescribing behaviour of cardiologists whom they consult. In addition, general practitioners may purposefully reduce the number of different drugs that they prescribe in order to learn more effectively about the benefits and risks of these drugs. Cardiologists tend to share patients with many other physicians and they tend to prescribe new cardiological medication earlier than primary care physicians, so the identified effects may not reflect network mechanisms only. However, the effects of high degree centrality were also found for general practitioners and family doctors, as they existed independently of physicians’ specialty. This supports the hypothesized network effects on medication prescribing decisions. Physicians’ mutual connections through shared patients may influence their opinions on prescribing ARNI, which may be explained by mechanisms such as social influence (e.g. imitation), opinion leadership, and dependency with respect to patient referrals. The strength of the effect of the network on prescribing in the observed 2 years may be described as small to moderate, but the accumulation over several years implies that it ultimately has a substantial impact.

Our results are in line with those of Donohue et al. [16] and Sundmacher et al. [24], who showed that physicians whose peers prescribed the new drug are more likely to adopt the new medication. In the classic study ‘Medical Innovation: A Diffusion Study’, Coleman et al. [25] describe that social interactions accelerate the adoption of tetracycline, a broad-spectrum antibiotic, in the first few months after market approval. Roger’s ‘diffusion of innovation’ theory [26] also points to the effect of social influence. Central actors play a major role in the adoption process. Through their many connections, they receive information more quickly and thus adopt new innovations earlier than less central actors. As measured by degree centrality, we showed that prescribers had more linkages in the network than non-prescribers. This is consistent with the findings of Iyengar et al. [27]. They showed the adoption of a new drug for treatment of viral infection. Prescribers had more incoming connections (high in-degree) with other physicians versus non-prescribers. The analysis took place in a network in which referrals form the links.

Some network mechanisms explain the increasing homogeneity of beliefs and behaviours in a network, but homogeneity itself does not necessarily imply a specific direction of change (e.g. increasing prescribing of a new drug). The latter requires a driving force, such as trusted clinical guidance or a trusted opinion leader. Having many connections is one feature of clinical opinion leadership [28, 29]. Valente and Yon [30] set in their simulation study the starting point for the spread of an innovation among physicians with high in-degree, who are referred to as opinion leaders. This led to a faster and farther spreading adoption of the innovation compared to a randomly selected starting point.

In our study, physicians who have many connections to other physicians who are not mutually connected, as measured by constraint, were more likely to be connected to physicians who prescribed the new drug than physicians who were strongly involved in the network. These low-constrained networks are more open to new information. According to Granovetter’s theory of weak ties [31], individuals on bridges of networks can bring new information into a network and thus promote for example the adoption of a new drug. In a closed network with a high constraint, there are no such persons and new information might not reach the network members. Such information can reach physicians at so-called quality circles [32], for example. Quality circles are meetings between physicians where freely selected topics are discussed in small groups and experiences are exchanged [33]. In a qualitative study of antibiotic prescribing behaviour, Poss-Doering et al. [34] showed that primary care networks can lead to more guideline-compliant prescribing behaviour through social influence and social support.

In contrast to degree centrality or constraint, betweenness centrality did not explain the uptake of ARNI. This could be explained by individuals with high betweenness centrality have power over others because they can control the flow of information between two people. Since this is not accompanied by social contagion, our theoretical considerations are confirmed.

Structured care models such as the disease management programme for coronary heart disease were included as a covariate in the analysis, because participation in this programme is more likely to lead to the prescription of the new drug than non-participation. Roehl et al. [35] show that physicians in such programmes are more likely to implement the recommendations of the guidelines than those who are not in the programme.

## Strength and limitations

A major strength of the study is the longitudinal observational design, in which predictors preceded outcomes in time. This design facilitates a causal interpretation of the correlations, although we cannot fully rule out confounding. We were able to quantify network effects in a large real-world study. A further strength of the study is the inclusion of the whole network of physicians in a large region through claims data from a health insurer. Moreover, and typical for claims data, the data is nearly complete with only a few missing values for location.

Nevertheless, a limitation of claims data is that it is not possible to verify the indication for prescribing the new medication, because there are only International Statistical Classification of Diseases and Related Health Problems (ICD-10) presented and no clinical data. For example, the ejection rate of the heart is not shown in the health insurance data. Also, our study did not include patient outcomes, such as the number of patients who benefit from a prescription of ARNI. There is a possibility that some physicians do not have the opportunity to prescribe the new drug or that they do not have patients who need the drug. Another possible confounder may be links to pharmaceutical companies. The data do not allow any statement on a possible influence by ‘advertising campaigns’. Finally, we used data from one health insurer only, which is known to underrepresent specific population groups such as citizens with high income and specific employees in public services who might be the first to be treated with this new drug. However, we expect that this only mildly affected our results, as nearly all physicians were included in our analysis, and on the physician level, no structural bias is to be expected. An analysis with the data from 2019 would also have been desirable, but the data were not available at the time of analysis.

## Conclusions

Our study provides evidence that physicians’ prescribing behaviour and uptake of a new drug are influenced by connections to other physicians via shared patients. Physicians with more connections to physicians who already prescribed ARNI were more likely to prescribe the combination of sacubitril/valsartan, independent of physicians’ specialty.

## Supplementary Information

**Additional file 1.** Reporting Guideline.

**Additional file 2.** Multivariable logistic regression: subgroup non-prescriber 2017.

## Data Availability

The data that support the findings of this study are available from AOK Baden-Wurttemberg but restrictions apply to the availability of these data, which were used under licence for the current study, and so are not publicly available. Data are however available from the authors upon reasonable request and with permission of AOK Baden-Wurttemberg.

## Abbreviations

AME: Average marginal effect
AOK: German: *Allgemeine Ortskrankenkasse*
ARNI: Angiotensin receptor and neprilysin inhibitor
CI: Confidence interval
DDD: Defined daily dose
DMP: Disease management programme chronic coronary heart disease
HF: Heart failure
HzV: German: *Hausarztzentrierte Versorgung* (family doctor-centred care)
ICD-10: 10th revision of the International Statistical Classification of Diseases and Related Health Problems
IQR: Interquartile range
NYHA: New York Heart Association
Ref.: Reference group
SD: Standard derivation
SE: Standard error
