# Supplementary material for: Influence of physician networks on prescribing a new ingredient combination in heart failure: a longitudinal claim data-based study
Source: Implement Sci. 2021 Aug 28;16:84. doi: 10.1186/s13012-021-01150-y (PMC8401102; doi:10.1186/s13012-021-01150-y)
Supplement: Supplementary file 2 — Additional file 2. Multivariable logistic regression: subgroup non-prescriber 2017. [file 13012_2021_1150_MOESM2_ESM.docx]

**Additional file 2: Multivariable logistic regression (subgroup non-prescriber 2017) dependent variable: ARNI prescription in 2018 (N= 5,796)**

| **Variable** | **AME** | **95 % CI** | **p-value** |
| --- | --- | --- | --- |
| Link to ARNI prescriber (ref. few 0–5 links)  Medium (6–10 links)  Many (> 10 links) | 0.03  0.07 | 0.003—0.06  0.04—0.11 | < 0.05  < 0.001 |
| Prescription ARNI 2017 (ref. no) | 0.39 | 0.37—0.40 | < 0.001 |
| Constraint (multiplied by 10) | –0.06 | –0.08— –0.04 | < 0.001 |
| Betweenness centrality (multiplied by 1000) | –0.001 | –0.004—0.002 | 0.38 |
| Specialists (ref. family-doctors) |  |  |  |
| General practitioners | –0.01 | –0.02—0.03 | 0.65 |
| Internal medicine | –0.18 | –0.30— –0.06 | 0.001 |
| Cardiologists | 0.07 | 0.004—0.13 | < 0.05 |
| Pneumologists | –0.44 | –0.67— –0.21 | < 0.001 |
| Angiologists | –2.10 | –54—50 | 0.94 |
| Nephrologists | –0.14 | –0.24— –0.04 | < 0.001 |
| Sex (ref. female) | 0.02 | 0.002—0.05 | < 0.05 |
| Urban-rural (ref. rural) | –0.04 | –0.07— –0.02 | < 0.001 |
| Family-doctors centred care (ref. no) | 0.05 | 0.02—0.07 | < 0.001 |
| Disease management program (ref. no) | 0.09 | 0.06—0.13 | < 0.001 |
| Number of patients with heart failure | 0.001 | 0.00—0.001 | < 0.001 |
| Total variance explained by the model (R²) | 0.32 | Cohen-effect | 0.68 |

AME= average marginal effect

CI= Confidence interval

Ref= reference group
